# ARF1 with Sec7 Domain-Dependent GBF1 Activates Coatomer Protein I To Support Classical Swine Fever Virus Entry

**DOI:** 10.1128/jvi.02193-21

**Published:** 2022-03-23

**Authors:** Liang Zhang, Tao Wang, Yanyan Yi, Mengzhao Song, Mingxing Jin, Kangkang Guo, Yanming Zhang

**Affiliations:** a College of Veterinary Medicine, Northwest A&F University, Yangling, Shaanxi, China; Instituto de Biotecnologia/UNAM

**Keywords:** classical swine fever virus, GBF1, ARF1, COP I, cholesterol trafficking

## Abstract

Classical swine fever virus (CSFV), a positive-sense, enveloped RNA virus that belongs to the *Flaviviridae* family, hijacks cell host proteins for its own replication. We previously demonstrated that Golgi-specific brefeldin A (BFA) resistance factor 1 (GBF1), a regulator of intracellular transport, mediates CSFV infection. However, the molecular mechanism by which this protein regulates CSFV proliferation remains unelucidated. In this study, we constructed a series of plasmids expressing GBF1 truncation mutants to investigate their behavior during CSFV infection and found that GBF1 truncation mutants containing the Sec7 domain could rescue CSFV replication in BFA- and GCA (golgicide A)-treated swine umbilical vein endothelial cells (SUVECs), demonstrating that the effect of GBF1 on CSFV infection depended on the activity of guanine nucleotide exchange factor (GEF). Additionally, it was found that ADP ribosylation factors (ARFs), which are known to be activated by the Sec7 domain of GBF1, also regulated CSFV proliferation. Furthermore, we demonstrated that ARF1 is more important for CSFV infection than other ARF members with Sec7 domain dependence. Subsequent experiments established the function of coatomer protein I (COP I), a downstream effector of ARF1 which is also required for CSFV infection by mediating CSFV invasion. Mechanistically, inhibition of COP I function impaired CSFV invasion by inhibiting cholesterol transport to the plasma membrane and regulating virion transport from early to late endosomes. Collectively, our results suggest that ARF1, with domain-dependent GBF1 Sec7, activates COP I to facilitate CSFV entry into SUVECs.

**IMPORTANCE** Classical swine fever (CSF), a highly contact-infectious disease caused by classical swine fever virus (CSFV) infecting domestic pigs or wild boars, has caused huge economic losses to the pig industry. Our previous studies have revealed that GBF1 and class I and II ARFs are required for CSFV proliferation. However, a direct functional link between GBF1, ARF1, and COP I and the mechanism of the GBF1-ARF1-COP I complex in CSFV infection are still poorly understood. Here, our data support a model in which COP I supports CSFV entry into SUVECs in two different ways, depending on the GBF1-ARF1 function. On the one hand, the GBF1-ARF1-COP I complex mediates cholesterol trafficking to the plasma membrane to support CSFV entry. On the other hand, the GBF1-ARF1-COP I complex mediates CSFV transport from early to late endosomes during the entry steps.

## INTRODUCTION

Classical swine fever (CSF), a highly infectious disease caused by classical swine fever virus (CSFV), is a disease that must be reported to the World Organization for Animal Health (1). CSFV is a positive-strand, enveloped RNA virus that belongs to the *Flaviviridae* family. The CSFV RNA genome, with one large open reading frame flanked by 5′ and 3′ untranslated regions, is directly translated into a large polyprotein with 3,898 amino acids. This polyprotein is cleaved by host proteases and virally encoded proteases into four structural proteins (C, E^rns^, E1, and E2) and eight nonstructural proteins (N^pro^, p7, NS2, NS3, NS4A, NS4B, NS5A, and NS5B) (2).

Coatomer protein I (COP I)-coated vesicles are composed of α, β, β′, γ, γ′, δ, ε, ζ, and ζ′ subunits, forming a cage-like lattice structure similar to that of clathrin-coated vesicles, which mediate protein trafficking from the Golgi apparatus to the endoplasmic reticulum (ER) and trafficking between cisternae within the Golgi apparatus. Thus, COP I is localized at the ER-to-Golgi intermediate compartment (ERGIC) and the Golgi apparatus, where it is abundantly present at the cisternal rims and enriched toward the *cis* side (3). Moreover, COP I-coated vesicles are also involved in membrane transport in the endocytic pathway and are present on endosomes for the formation of vesicles that mediate endosomal transport from early to late endosomes (4). COP I-coated vesicle assembly is regulated by ADP ribosylation factors (ARFs) such as ARF1, a small GTPase of the Ras superfamily which must be activated into its GTP-bound state by the nucleotide exchange factor Golgi-specific brefeldin A (BFA) resistance factor 1 (GBF1). The Sec7 domain of GBF1 is a guanine nucleotide exchange factor (GEF) that activates the GDP/GTP exchange of class I (ARF1 to -3) and class II (ARF4 and -5) ARFs (5). ARF1 cycles between an inactive and an active GDP-bound form. The ARF1-T31N mutant mimics GDP-bound states, an inactive form of ARF1. ARF1-Q71I mimics GTP-bound states, an active form of ARF1 (6).

The GBF1-ARF1-COP I axis has recently been reported to play a regulatory role in viral entry, genome replication, viral protein transport, and progeny viral assembly for many families, including the *Picornaviridae* (7, 8), *Coronaviridae* (9), and *Flaviviridae* (10, 11). For hepatitis C virus (HCV), from the same *Flaviviridae* family as CSFV, inhibition of GBF1-ARF1-COP I impaired viral genome replication. Regarding the mechanism of replication inhibition, there is evidence that GBF1-ARF1-COP I could mediate Phosphatidylinositol-4-phosphate (PI4P) accumulation in HCV replication complexes to support HCV RNA replication (12). For dengue virus (DENV), another member of the *Flaviviridae* family, treatment with BFA, a GTP exchange factor inhibitor that blocks COP I activity, decreased virus production. In contrast to the mechanism that regulates HCV proliferation, the GBF1-ARF1-COP I system is used for the transfer of the capsid protein of DENV to lipid droplets to support viral assembly (13). These reports demonstrated that the GBF1-ARF1-COP I system supports viral proliferation by regulating the formation of the viral RNA replication complex and the transport of viral proteins, which is dependent on the transport function of COP I-coated vesicles. Additionally, in several viruses, entry into the host cell has been found to involve COP I, which is dependent on the role of COP I-coated vesicles in endosome assembly (14). However, certain viruses also utilize GBF1 in an ARF1-independent manner or utilize ARF1 in a GBF1-independent manner to promote replication (15, 16).

Our previous studies have revealed that GBF1 and class I and II ARFs are required for CSFV proliferation (17). However, a direct functional link between GBF1, ARF1, and COP I and the mechanism of the GBF1-ARF1-COP I axis in CSFV infection regulation have not been experimentally revealed. Here, we demonstrated the function of different GBF1 truncation mutants in CSFV infection and found that CSFV utilized ARF1 in a GBF1 Sec7 domain-dependent manner for infection. Furthermore, we report that COP I, as an effector of ARF1, is an essential host factor for CSFV proliferation. By determining the underlying mechanism of GBF1-ARF1-COP I function, we provide evidence that the GBF1-ARF1-COP I axis is required for CSFV entry by mediating intracellular cholesterol trafficking and virion endosomal transport.

## RESULTS

### The function of the GBF1 Sec7 domain in CSFV infection.

**FIG 1:**
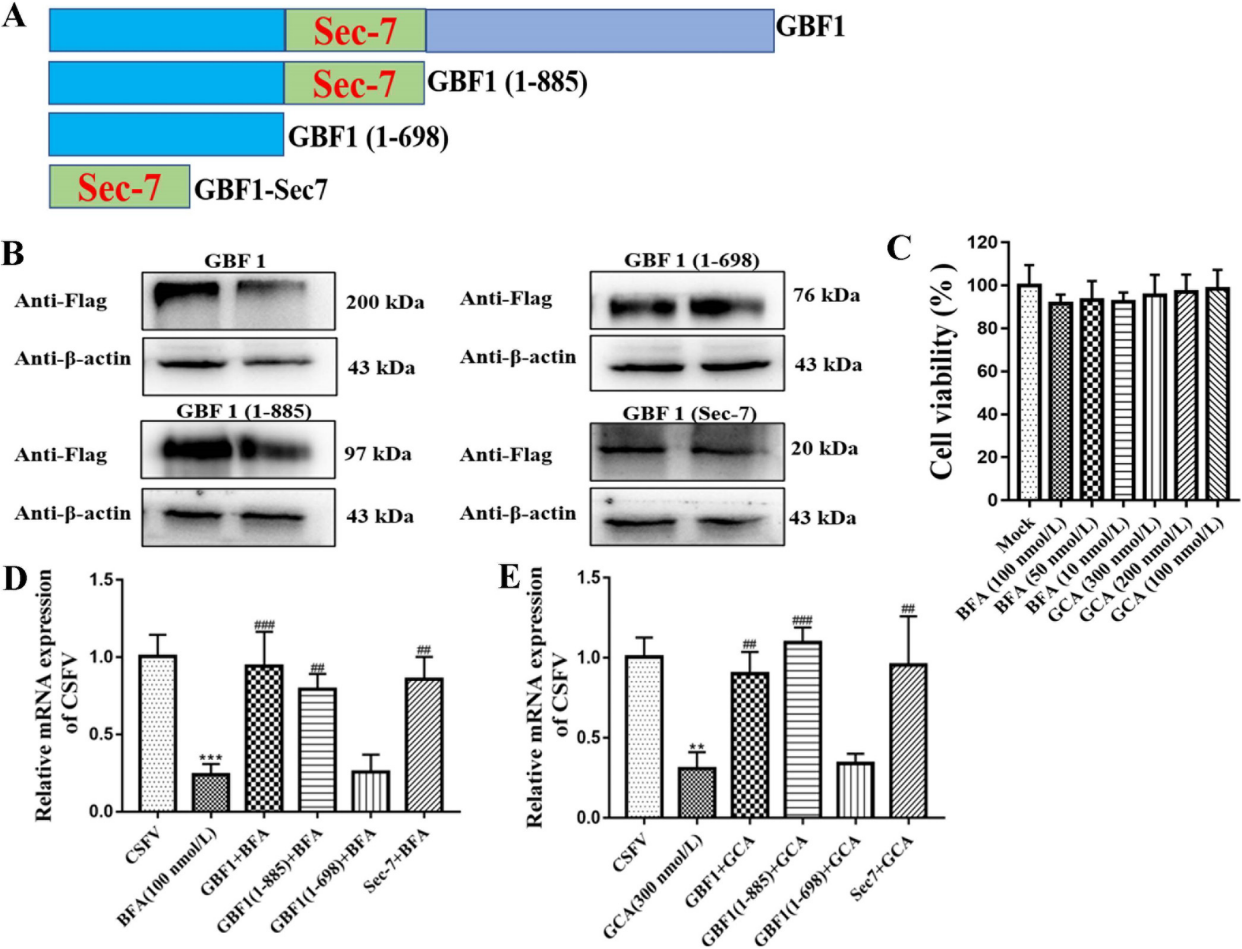
The Sec7 domain of GBF1 is required for CSFV production. (A) Diagram of GBF1 truncation mutants. (B) SUVECs were transfected with the indicated GBF1 plasmids [GBF1-FLAG, GBF1-(1–885)-FLAG, GBF1-(1–698)-FLAG, and GBF1-Sec7-FLAG], and cells were collected after transfection for 48 h. Next, protein samples were extracted with RIPA lysis, and GBF1-FLAG, GBF1-(1–885)-FLAG, GBF1-(1–698)-FLAG, and GBF1-Sec7-FLAG expression levels were measured by a Western blot assay with anti-FLAG antibody. β-Actin served as an internal control. (C) Cell viability upon treatment with BFA and GCA was measured by CCK-8 assays. (D and E) SUVECs were transfected with the indicated plasmids [GBF1-FLAG, GBF1-(1–885)-FLAG, GBF1-(1–698)-FLAG, and GBF1-Sec7-FLAG] for 48 h and cotreated with CSFV (MOI of 1) and BFA or GCA for 48 h. Next, cells were collected for RNA extraction with TRIzol lysis, and CSFV RNA copy numbers were measured by RT-qPCR. “*” represents the comparison between the BFA- or GCA-treated cells and the CSFV-infected cells, and “#” represents the comparison between the cells transfected with the indicated plasmids and the BFA- or GCA-treated cells.

GBF1, as a host factor, is required for CSFV infection of swine umbilical vein endothelial cells (SUVECs), as previously reported (17). However, the molecular mechanism of GBF1 function in CSFV infection is yet to be fully elucidated. Here, we further examined whether the presence of different GBF1 domains assisted in CSFV infection. First, full-length porcine GBF1 with a FLAG-tagged expression plasmid was created, and a series of expression plasmids for the GBF1 truncation mutants was also generated: the GBF1-(1–885)-FLAG plasmid with the C terminus removed, the GBF1-(1–698)-FLAG plasmid with the C terminus and Sec7 domain removed, and the GBF1-Sec7-FLAG plasmid with the C terminus and N terminus removed (Fig. 1A). These plasmids were transfected into SUVECs, and the expression of FLAG-tagged wild-type and mutant GBF1 proteins was confirmed by a Western blot assay (Fig. 1B). In addition, we also assessed the cytotoxicity of two GBF1 inhibitors, namely, BFA and GCA (golgicide A). The results of the cell viability assay showed that 100 nmol/L BFA and 300 nmol/L GCA demonstrated no obvious toxicity to the cells (Fig. 1C). Second, SUVECs were transfected with the indicated GBF1 plasmid [GBF1-FLAG, GBF1-(1–885)-FLAG, GBF1-(1–698)-FLAG, and GBF1-Sec7-FLAG] for 48 h, and cells were cotreated with CSFV (multiplicity of infection [MOI] of 1) and BFA (100 nmol/L). After 48 h, the cells were collected for viral genome copy number quantification. Our results showed that CSFV RNA copy numbers were significantly decreased in BFA-treated cells compared to those in CSFV-infected cells (*P < *0.05) (Fig. 1D), and viral genome copy numbers were partially rescued in GBF1-FLAG-, GBF1-(1–885)-FLAG-, and GBF1-Sec7-FLAG-transfected cells (*P < *0.05) (Fig. 1D). Unlike Sec7 domain-containing plasmids, GBF1-(1–698)-FLAG plasmid transfection did not rescue CSFV replication (*P > *0.05) (Fig. 1D). To verify the role of the GBF1 Sec7 domain in CSFV replication, we further examined the effect of GCA, a specific inhibitor targeting the GBF1 Sec7 domain, on CSFV replication. SUVECs were treated as described above, and similar results were obtained. GCA potently suppressed CSFV replication (*P < *0.05); however, the Sec7 domain-containing plasmid significantly rescued GCA-induced CSFV replication inhibition (*P < *0.05) (Fig. 1E). Taken together, our results reveal that the Sec7 domain of GBF1 is a key factor in CSFV proliferation.

### ARF1 was involved in CSFV infection in a Sec7 domain-dependent manner.

**FIG 2:**
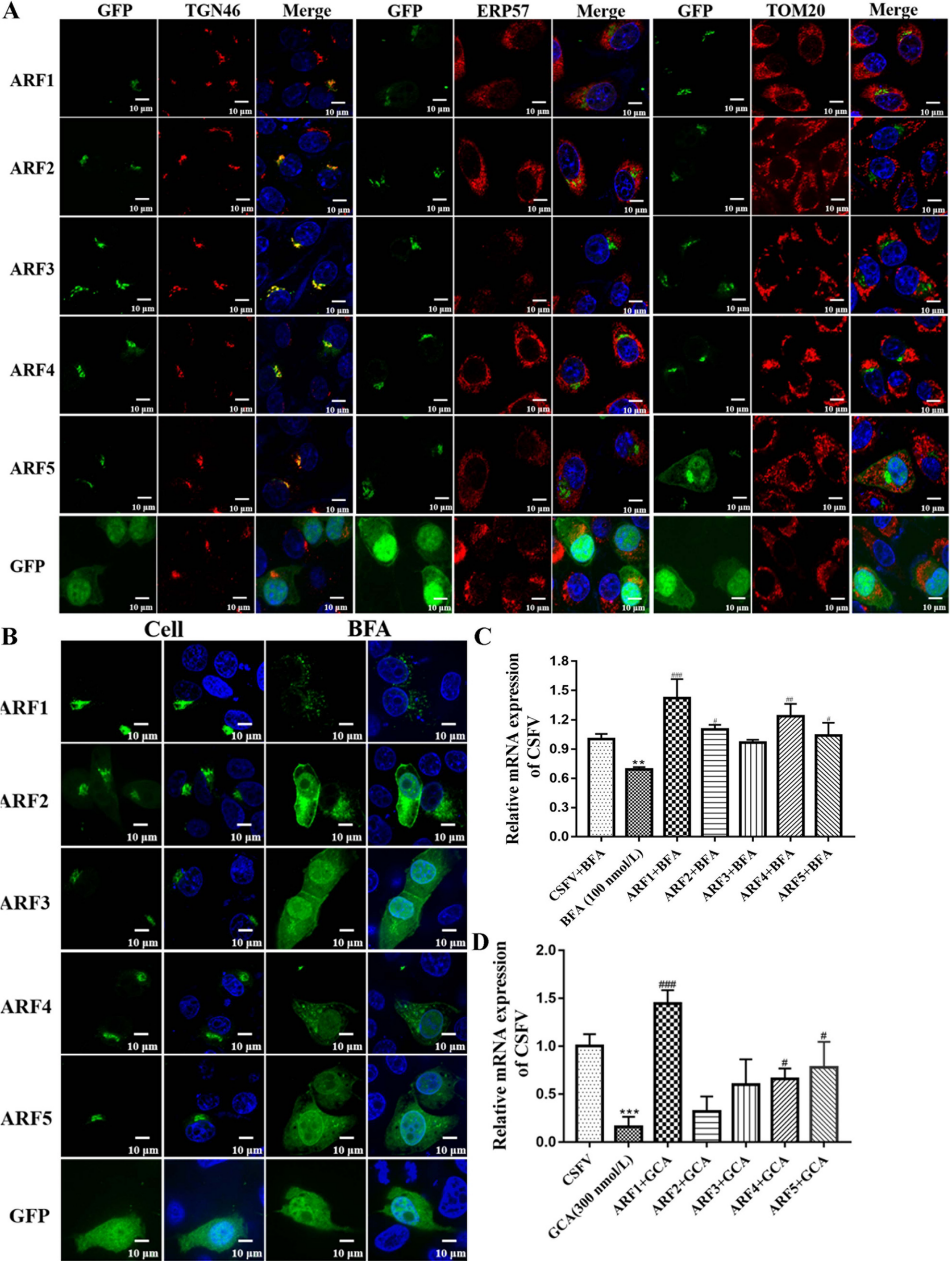
ARF1 is involved in CSFV infection in a GBF1 Sec7 domain-dependent manner. (A and B) Characterization of porcine ADP ribosylation factors (classes I and II). (A) SUVECs were seeded onto glass coverslips and transfected with the pEGFP-ARF1, pEGFP-ARF2, pEGFP-ARF3, pEGFP-ARF4, or pEGFP-ARF5 plasmid. Forty-eight hours after transfection, cells were fixed in 4% paraformaldehyde, and immunofluorescence staining was performed with anti-TGN46, ERP57, or TOM20. Cells were also counterstained with DAPI to label nuclei (blue). GFP, green fluorescent protein. (B) SUVECs were seeded onto glass coverslips and transfected with the pEGFP-ARF1, pEGFP-ARF2, pEGFP-ARF3, pEGFP-ARF4, or pEGFP-ARF5 plasmid. Forty-eight hours after transfection, cells were treated with or without BFA for 2 h. Next, cells were fixed in 4% paraformaldehyde and stained with DAPI to label nuclei (blue). (C and D) SUVECs were transfected with the indicated plasmids (pEGFP-ARF1, pEGFP-ARF2, pEGFP-ARF3, pEGFP-ARF4, and pEGFP-ARF5) for 48 h and cotreated with CSFV (MOI of 1) and BFA or GCA for 48 h. Next, cells were collected for RNA extraction with TRIzol lysis, and CSFV RNA copy numbers were measured by RT-qPCR. “*” represents the comparison between the BFA- or GCA-treated cells and the CSFV-infected cells, and “#” represents the comparison between the cells transfected with the indicated plasmids and the BFA- or GCA-treated cells.

GBF1 is a BFA-sensitive GEF for G proteins from the ARF family. The Sec7 domain has GEF activity, which activates class I and II ARFs (5, 18). Since the Sec7 domain of GBF1 is involved in the replication of CSFV in SUVECs, the function of Sec7 domain effector ARFs (class I and class II) in CSFV infection is also worthy of attention. To determine whether the Sec7 domain promoted CSFV infection through the function of ARFs, we cloned the ARF1 to ARF5 genes into the pEGFP-N1 vector and verified their expression in transfected cells using Western blotting (data not shown). Following the characterization of the expression of ARFs in cells, the subcellular distribution of each of the ARFs was demonstrated in transfected cells. Using confocal microscopy, we observed that class I (ARF1, ARF2, and ARF3) and class II (ARF4 and ARF5) ARFs were located in the Golgi complex but not in the ER or mitochondria, indicating their mediator function in Golgi association trafficking (Fig. 2A). Furthermore, we screened which porcine ARF function is Sec7 domain dependent. SUVECs were transfected with the indicated plasmids and treated with or without BFA for 2 h. The subcellular distribution of different ARFs was disrupted with 100 nmol/L BFA as shown by using confocal microscopy (Fig. 2B). In contrast, pEGFP-ARF1 to pEGFP-ARF5, but not pEGFP, clustered around the nucleus in untreated cells (Fig. 2B). This result revealed that the porcine ARF1 to ARF5 proteins were sensitive to BFA, indicating that the functions of all ARFs were Sec7 domain dependent. Considering that all ARF functions were Sec7 domain dependent in SUVECs, we speculated that the activation of at least one ARF family member is required for CSFV replication. To this end, SUVECs were transfected with pEGFP-ARF1, pEGFP-ARF2, pEGFP-ARF3, pEGFP-ARF4, or pEGFP-ARF5 for 48 h and then cotreated with CSFV, BFA, or GCA for 48 h. We observed that ARF1 markedly rescued CSFV replication in Sec7 domain activity-defective cells (*P < *0.05) (Fig. 2C and D). Together, these data suggest that ARF1 plays a major GBF1 Sec7-dependent role in CSFV replication.

### ARF1 positively regulated CSFV propagation.

**FIG 3:**
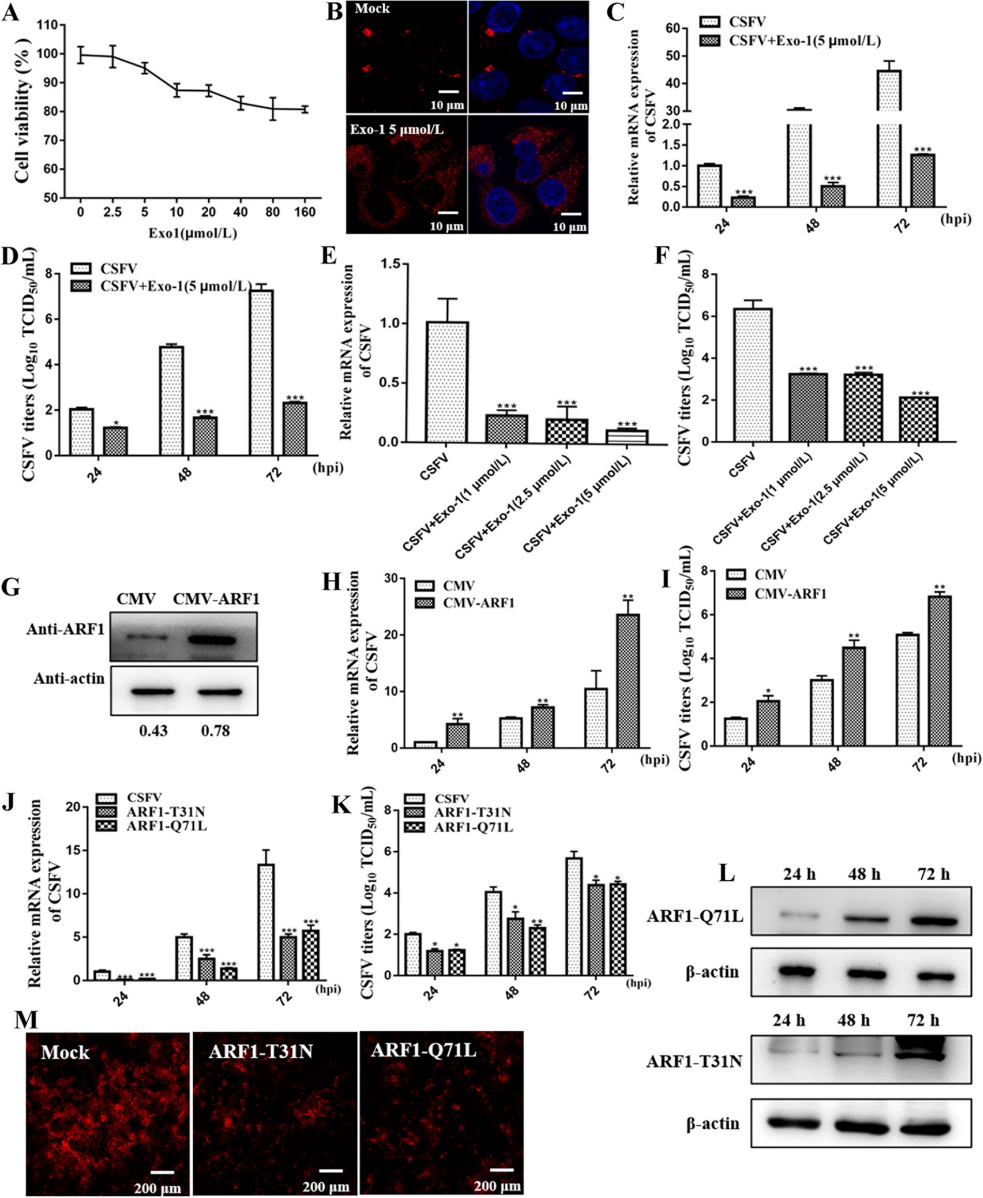
ARF1 positively regulates CSFV propagation. (A to F) Exo-1 treatment decreases CSFV production. (A) Cell viability upon treatment with Exo-1 was measured by CCK-8 assays. (B) SUVECs were seeded onto glass coverslips and treated with or without Exo-1 for 2 h. Next, cells were fixed in 4% paraformaldehyde, and immunofluorescence staining was performed using an anti-TGN46 antibody. Cells were also counterstained with DAPI to label nuclei (blue). (C to F) SUVECs were cotreated with CSFV (MOI of 1) and 5 μmol/L Exo-1 or various concentrations of Exo-1 (5, 2.5, or 1 μmol/L), and cell pellets and culture supernatants were harvested for CSFV RNA copy number and CSFV viral titer determinations by RT-qPCR and TCID_50_ per milliliter at the indicated time points. hpi, hours postinfection. (G) Confirmation of CMV and CMV-ARF1 cell lines by detection of ARF1 protein expression by Western blotting. β-Actin served as an internal control. (H and I) CMV and CMV-ARF1 cell lines were infected with CSFV (MOI of 1). At 24, 48, and 72 h, cell pellets and culture supernatants were harvested for CSFV RNA copy number and CSFV viral titer determinations by RT-qPCR and TCID_50_ per milliliter. (J and K) SUVECs were transfected with pEGFP-ARF1-T31N or pEGFP-ARF1-Q71L, and cells were infected with CSFV (MOI of 1) after 24 h of transfection. At 24, 48, and 72 h, cell pellets and culture supernatants were harvested for CSFV RNA copy number and CSFV viral titer determinations by RT-qPCR and TCID_50_ per milliliter. (L) SUVECs were transfected with pEGFP-ARF1-T31N and pEGFP-ARF1-Q71L. After 24 h of transfection, cells were infected with CSFV (MOI of 1). Next, cells were collected for the determination of ARF1-T31N and ARF1-Q71L protein expression by Western blot assays at the indicated time points. β-Actin served as an internal control. (M) Confocal microscopy analyses of CSFV replication in pEGFP-ARF1-T31N- and pEGFP-ARF1-Q71L-transfected cells. SUVECs were transfected with pEGFP-ARF1-T31N or pEGFP-ARF1-Q71L. After 48 h of transfection, cells were infected with CSFV (MOI of 1) for 24 h. Next, cells were fixed in 4% paraformaldehyde and stained with anti-E2 antibody (red).

To investigate the involvement of ARF1 in regulating CSFV infection, we examined the effect of Methyl 2-(4-fluorobenzamido)benzoate Exo-1, a specific inhibitor of ARF1, on CSFV propagation. A cell counting kit 8 (CCK-8) assay revealed that the safe concentration of Exo-1 for SUVECs was 5 μmol/L (Fig. 3A). We also observed that Exo-1, at a concentration of 5 μmol/L, caused Golgi structure fragments as seen under a confocal microscope (Fig. 3B), indicating that Exo-1 at 5 μmol/L could disrupt the function of ARF1. Using reverse transcription-quantitative PCR (RT-qPCR) and 50% tissue culture infectious doses (TCID_50_) per milliliter, we observed a dose- and time-dependent restriction of CSFV proliferation by Exo-1 (*P < *0.05) (Fig. 3C to F). Next, stably ARF1-overexpressing (CMV-ARF1) cell lines were obtained by using recombinant lentiviruses and were utilized to functionally validate the correlation between ARF1 and CSFV infection. Our results showed that ARF1 overexpression promoted CSFV proliferation (*P < *0.05) (Fig. 3G to I).

In light of the findings of the role of ARF1 in CSFV infection, we investigated whether viral production was also mediated by ARF1-T31N and ARF1-Q71L. The ARF1-T31N mutant mimics GDP-bound states, an inactive form of ARF1. ARF1-Q71I mimics GTP-bound states, an active form of ARF1. To this end, pEGFP-ARF1-T31N- and pEGFP-ARF1-Q71L-transfected cells were infected with CSFV (MOI of 1). After 24, 48, and 72 h of infection, as depicted in Fig. 3J and K, viral genome copy numbers and infectious viral proliferation were decreased in cells expressing pEGFP-ARF1-T31N or pEGFP-ARF1-Q71L (*P < *0.05). Moreover, CSFV proliferation in pEGFP-ARF1-T31N- or pEGFP-ARF1-Q71L-transfected cells was verified using an indirect immunofluorescence assay (IFA) 24 h after CSFV infection. The ARF1-T31N and ARF1-Q71L protein expression levels in transfected cells were determined using Western blotting at the indicated times (Fig. 3L). The CSFV plaques in pEGFP-ARF1-T31N- or pEGFP-ARF1-Q71L-transfected cells were smaller and less abundant than those in the control cells (Fig. 3M). These results strongly support the positive role of ARF1 in CSFV replication.

### COP I depletion inhibited CSFV invasion.

**FIG 4:**
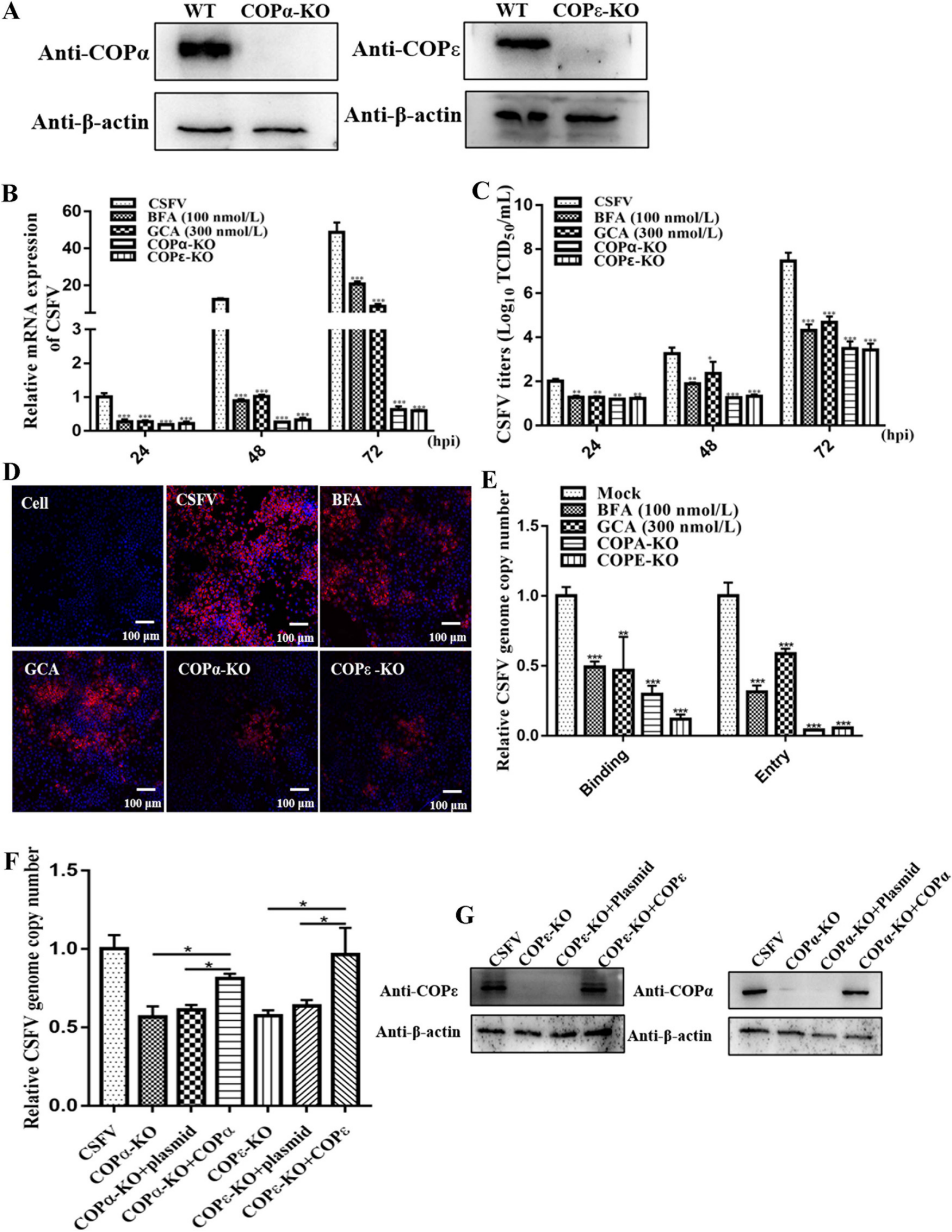
COP I was required for CSFV invasion. (A) Western blot analyses of COPα and COPε protein expression in COPα and COPε knockout cell lines. β-Actin served as an internal control. WT, wild type. (B and C) RT-qPCR and TCID_50_ per milliliter analyses of CSFV RNA copy numbers and CSFV viral titers in COPα and COPε knockout cells and cell supernatants. COPα and COPε knockout cell lines were infected with CSFV (MOI of 1), and SUVECs were cotreated with CSFV (MOI of 1) and an inhibitor (BFA or GCA). At 24, 48, and 72 h, cell pellets and culture supernatants were harvested for CSFV RNA copy number and CSFV viral titer determinations by RT-qPCR and TCID_50_ per milliliter. (D) Confocal microscopy analyses of CSFV replication in COPα and COPε knockout cell lines and BFA- and GCA-treated cells. Cell lines were infected with CSFV (MOI of 1), and SUVECs were cotreated with CSFV (MOI of 1) and an inhibitor (BFA or GCA) for 24 h. Next, cells were fixed in 4% paraformaldehyde and stained with anti-E2 antibody (red). (E) SUVECs were pretreated with BFA (100 nmol/L) or GCA (300 nmol/L) for 24 h. Next, inhibitor-treated cells and COPα and COPε knockout cell lines were infected with CSFV (MOI of 10) for 1 h at 4°C. Next, unbound virions were washed away using a precooled citrate buffer solution (pH 3), and cell pellets were harvested for CSFV RNA copy number determination by RT-qPCR to measure CSFV binding. SUVECs were pretreated with different concentrations of BFA (10, 50, and 100 nmol/L) and GCA (100, 200, and 300 nmol/L) for 2 h, and cells were incubated with CSFV (MOI of 10) for 1 h at 4°C. Next, cells were washed with PBS and cultured in fresh medium at 37°C for 2 h, and cell pellets were harvested for CSFV genome copy number determination by RT-qPCR to measure CSFV entry. (F and G) COPα and COPε knockout cell lines were transfected with pcDNA-COPα and pcDNA-COPε. At 48 h, cells were infected with CSFV (MOI of 10), the expression levels of COPα and COPε were measured by Western blotting, and virus entry was determined as described above for panel E.

COP I is a major substrate of ARF1, which coordinates cell component trafficking through vesicles in the early secretory pathway. Previous studies have demonstrated that COP I, ARF1, and GBF1 act as a complex to mediate cellular functions (19). Given that ARF1 functions rely on the GBF1 Sec7 domain to participate in CSFV proliferation, we investigated whether COP I depends on the GBF1-ARF1 pathway to mediate CSFV infection. We utilized the CRISPR/Cas9 gene-editing system to generate COP I subunit knockout (KO) cell lines, designated COPα-KO and COPε-KO. COPα and COPε knockouts were verified using Western blotting (Fig. 4A). Next, the COPα-KO and COPε-KO cell lines were infected with CSFV at an MOI of 1; cells treated with BFA (100 nmol/L) and GCA (300 nmol/L), which are GBF1 inhibitors, served as controls. After 24, 48, and 72 h of infection, cells were collected for viral genome copy number quantification, whereas the supernatants were collected for viral titration. The results showed that the genetic depletion of COP I expression resulted in a significant decrease in CSFV replication as well as in BFA (100 nmol/L)- and GCA (300 nmol/L)-treated cells (Fig. 4B and C) (*P < *0.05). Moreover, CSFV proliferation in cell lines was examined using an indirect IFA 24 h after CSFV infection. The CSFV plaques of the COPα-KO and COPε-KO cell lines and BFA (100 nmol/L)- and GCA (300 nmol/L)-treated cells were smaller and less abundant than those of the control cells (Fig. 4D). Collectively, our findings suggest that COP I knockout inhibits CSFV proliferation.

Given the role of COP I in CSFV infection, we conducted experiments to determine which steps of the CSFV life cycle were COP I mediated. First, COPα-KO and COPε-KO cell lines and BFA (100 nmol/L)- and GCA (300 nmol/L)-pretreated cells were used to determine the effect of COP I on CSFV binding and entry. To determine viral binding, SUVECs were pretreated with 100 nmol/L BFA and 300 nmol/L GCA for 24 h. Next, COPα-KO and COPε-KO cell lines and BFA- and GCA-treated cells were inoculated with CSFV in fetal bovine serum (FBS)-free medium for 1 h at 4°C. Unbound virions were then washed away using a precooled citrate buffer solution (pH 3). Total cells were collected for RNA extraction to determine the CSFV genome copy numbers. The results showed that the CSFV genome copy number was decreased in COPα-KO and COPε-KO cell lines and BFA- and GCA-treated cells, suggesting that COP I was required for CSFV binding (*P < *0.5) (Fig. 4E). To determine viral entry, SUVECs were pretreated with 100 nmol/L BFA and 300 nmol/L GCA for 24 h. Next, COPα-KO and COPε-KO cell lines and BFA- and GCA-treated cells were infected with CSFV in FBS-free medium for 1 h at 4°C to allow virion binding. Cells were then washed with a precooled citrate buffer solution (pH 3) to remove unbound virions and cultured for another 2 h at 37°C. The cells were extensively washed and collected for RNA isolation to determine the CSFV genome copy numbers. The results showed that CSFV genome copy numbers were decreased in COPα-KO and COPε-KO cell lines and BFA- and GCA-treated cells, suggesting that COP I was required for CSFV entry (*P < *0.5) (Fig. 4E). To further clarify the result that COPα and COPε knockout could inhibit the invasion of CSFV, COPα and COPε knockout cells were transfected with the recombinant plasmid expressing the corresponding protein, and the entry of CSFV was then detected. The results showed that target protein expression could partially reduce the suppression of CSFV invasion caused by COPα and COPε knockout (*P < *0.05) (Fig. 4F and G). Altogether, these results demonstrate the involvement of COP I in CSFV invasion.

### COP I inhibition impaired CSFV invasion by disrupting cholesterol transport to the plasma membrane.

**FIG 5:**
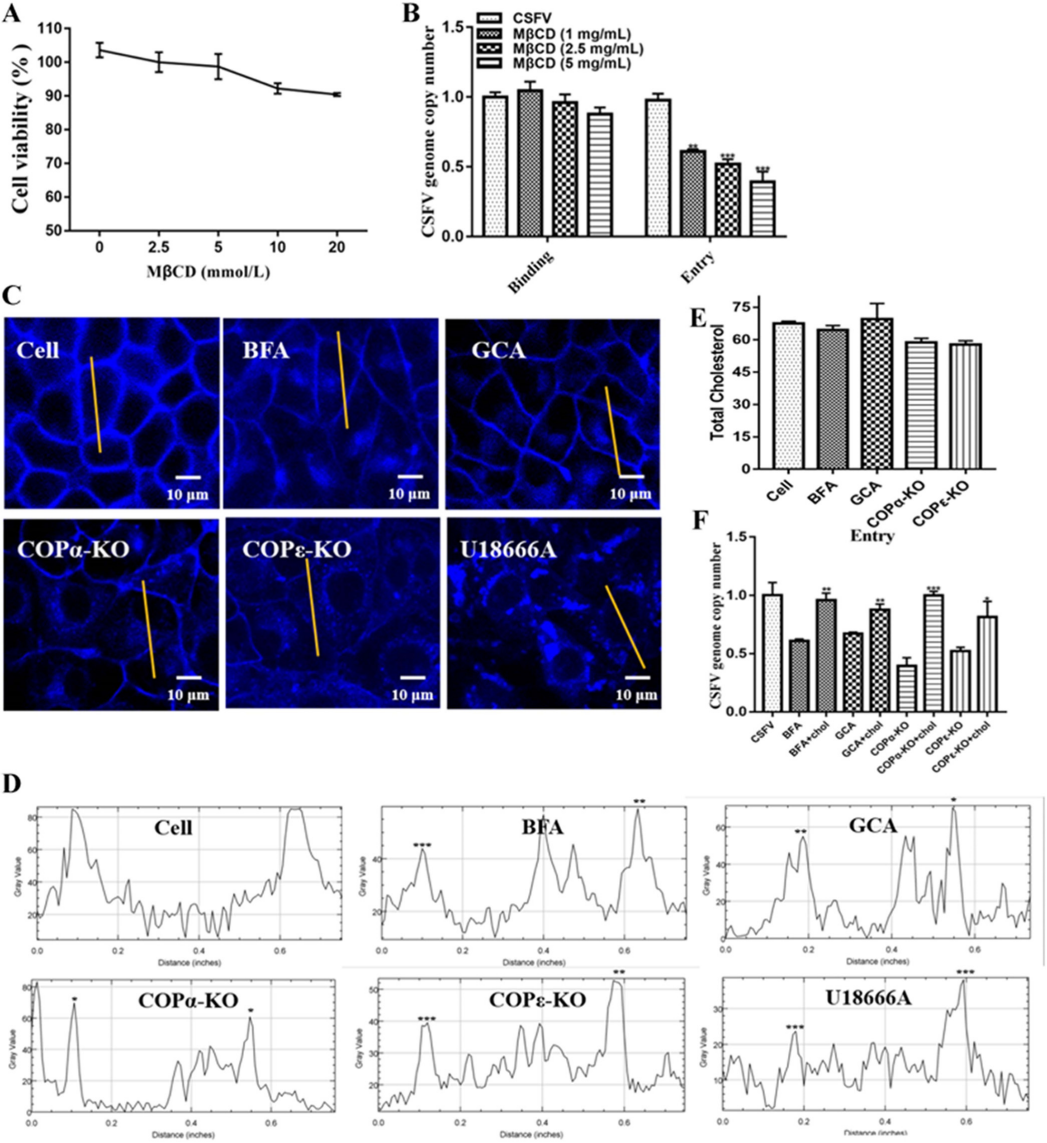
COP I inhibition impairs CSFV entry through disrupting cholesterol trafficking. (A) Cell viability upon treatment with MβCD was measured by CCK-8 assays. (B) SUVECs were pretreated with various concentrations of MβCD (5, 2.5, or 1 mg/mL) for 12 h and incubated with CSFV for 1 h at 4°C, unbound virions were washed away using a precooled citrate buffer solution (pH 3), and cell pellets were harvested for CSFV genome copy number determination by RT-qPCR to measure CSFV binding. SUVECs were pretreated with 5 mg/mL MβCD for 12 h, and cells were incubated with CSFV for 1 h at 4°C. Next, cells were washed with a precooled citrate buffer solution (pH 3) and cultured in the fresh medium at 37°C for 2 h, and cell pellets were harvested for CSFV genome copy number determination by RT-qPCR to measure CSFV entry. (C and D) Quantification of the cholesterol signal on the membrane relative to the cytosol. BFA (100 nmol/L)- and GCA (300 nmol/L)-treated SUVECs (for 8 h) and COPα and COPε knockout cells were fixed, and cholesterol was labeled by filipin. The intracellular cholesterol distribution was measured by confocal microscopy and quantified by using ImageJ. (E) BFA (100 nmol/L)- and GCA (300 nmol/L)-treated SUVECs (for 8 h) and COPα and COPε knockout cells were harvested for total cholesterol determination by a Cholesterol/Cholesterol Ester-Glo assay kit from Promega. (F) BFA (100 nmol/L)- and GCA (300 nmol/L)-treated SUVECs (for 8 h) and COPα and COPε knockout cells were treated with cholesterol for 2 h, and cells were incubated with CSFV for 1 h at 4°C. Next, cells were washed with a precooled citrate buffer solution (pH 3) and cultured in fresh medium at 37°C for 2 h, and cell pellets were harvested for CSFV genome copy number determination by RT-qPCR to measure CSFV entry.

Previous studies have demonstrated that ARF1, which activates the assembly of COP I vesicles, mediates cell cholesterol trafficking (19, 20). Intriguingly, a recent study on CSFV infection of PK-15 cells revealed that plasma membrane cholesterol is required for CSFV invasion (21). Thus, we speculated that COP I was required for CSFV invasion by mediating cholesterol trafficking. We first ensured that the results showing that methyl-β-cyclodextrin (MβCD), which is primarily used as a cholesterol-depleting reagent, inhibits CSFV entry by impairing plasma membrane cholesterol were not cell specific. SUVECs were pretreated with different concentrations of MβCD for 12 h, and the influence of MβCD on CSFV binding and entry was evaluated as described above. As shown in Fig. 5A and B, we found that MβCD dramatically reduced viral entry in SUVECs in a dose-dependent manner (*P < *0.05). Next, we determined whether COP I could regulate cholesterol trafficking by incubating cells with BFA and GCA or using COPα and COPε knockout cell lines. Cells were stained with filipin, which labels unesterified cholesterol, at room temperature for 30 min, and confocal laser microscopy revealed that cholesterol was mainly distributed on the cell membrane in control cells, whereas cholesterol was mainly concentrated in the perinuclear area in BFA- and GCA-treated cells and COPα and COPε knockout cells (Fig. 5C). Importantly, cholesterol was not present at the plasma membrane after COP I inactivation (Fig. 5D). To rule out the possibility that COP I depletion may affect the content of cholesterol in the cells, the total cholesterol contents in control cells and COP I-depleted cells were analyzed, and the results showed that COP I depletion did not affect the cell cholesterol content (*P > *0.05) (Fig. 5E). Moreover, the invasion efficiency of CSFV could be partially restored by replenishing cholesterol in COP I-inactivated cells (Fig. 5F). Together, these results demonstrate that COP I depletion inhibits CSFV invasion by disrupting cholesterol trafficking to the plasma membrane.

### COP I depletion inhibited endosomal trafficking upon virus internalization.

**FIG 6:**
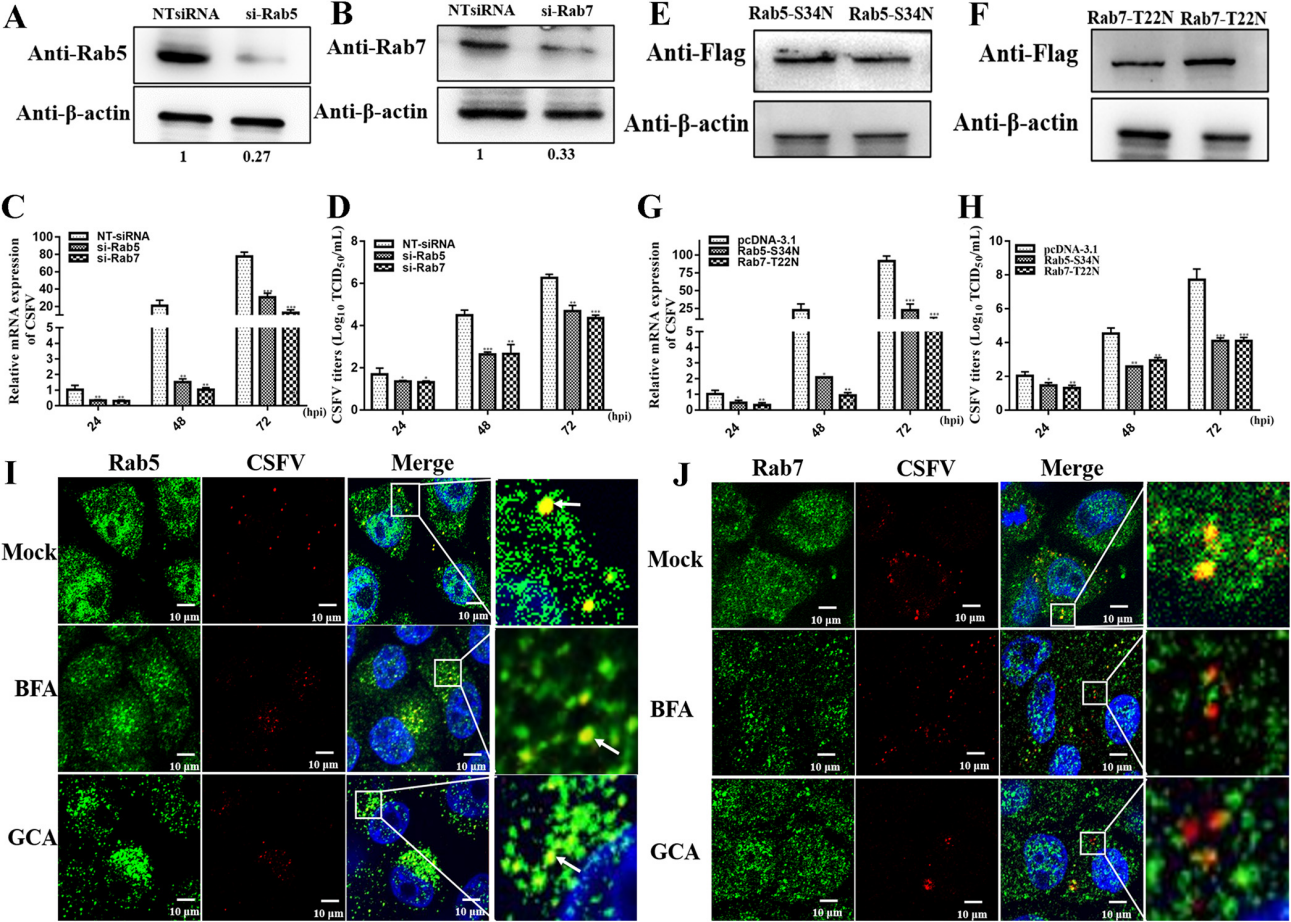
COP I depletion inhibits endosomal trafficking. (A and B) SUVECs were transfected with siRab5 or siRab7, and cells were harvested after 48 h of transfection. Next, the protein expression levels of Rab5 and Rab7 were determined by Western blotting. (C and D) SUVECs were transfected with siRab5 or siRab7, and cells were infected with CSFV (MOI of 1) after 48 h of transfection. At 24, 48, and 72 h, cell pellets and culture supernatants were harvested for CSFV genome copy number and CSFV viral titer determinations by RT-qPCR and TCID_50_ per milliliter. (E and F) SUVECs were transfected with Rab5-S34N or Rab-T22N, and cells were harvested after 48 h of transfection. Next, the protein expression level of Rab5-S34N or Rab-T22N was determined by Western blotting. (G and H) SUVECs were transfected with Rab5-S34N or Rab-T22N, and cells were infected with CSFV (MOI of 1) after 24 h of transfection. At 24, 48, and 72 h, cell pellets and culture supernatants were harvested for CSFV genome copy number and CSFV viral titer determinations by RT-qPCR and TCID_50_ per milliliter. (I and J) SUVECs were pretreated with 100 nmol/L BFA or 300 nmol/L GCA for 8 h, and cells were infected with CSFV (MOI of 1) for 2 h. Next, cells were stained with anti-E2 antibody, and the colocalization of CSFV particles with Rab5 or Rab7 was measured by confocal microscopy.

CSFV enters early endosomes and then fuses to late endosomes to enter porcine kidney 15 (PK-15) cells and porcine alveolar macrophages (PAMs) (22, 23). Furthermore, previous studies have reported that COP I proteins may be important for endosomal trafficking (4). However, it remains unclear thus far whether COP I vesicles directly mediate the sorting of CSFV between different types of endosomes in the invasion step. Thus, we first used Rab5 and Rab7 small interfering RNAs (siRNAs) to investigate the role of early or late endosomes in CSFV infection. SUVECs were transfected with siRNA targeting Rab5 (siRab5) or siRab7, and the silencing efficiency was determined using Western blotting (*P < *0.05) (Fig. 6A and B). Next, siRNA-transfected cells were infected with CSFV (MOI of 1), and the results showed that compared to nontargeting siRNA (NTsiRNA)-transfected cells, viral RNA copy numbers and the proliferation of CSFV were significantly decreased in Rab5- and Rab7-silenced cells (*P < *0.05) (Fig. 6C and D).

**FIG 7:**
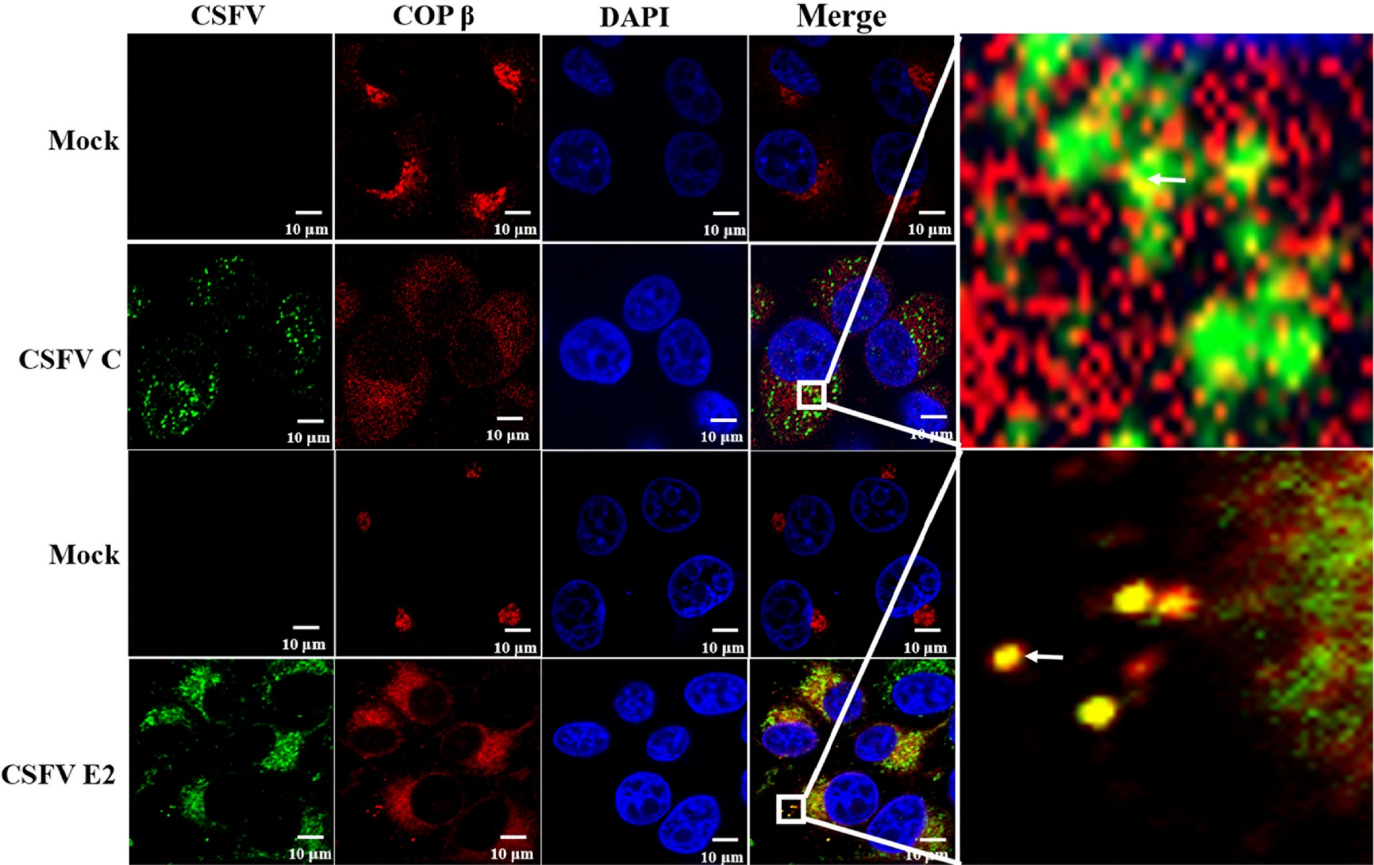
CSFV core and E2 proteins colocalized with COP I. SUVECs were infected with CSFV (MOI of 1) for 12 h and fixed in 4% paraformaldehyde. Next, cells were costained with anti-E2 and anti-COPβ antibodies or costained with anticore and anti-COPβ antibodies.

We examined the expression of Rab5(S34N) and Rab7(T22N) to further confirm the early and late endosomes in CSFV infection. The expression levels of Rab5(S34N) and Rab7(T22N) in transfected SUVECs were investigated using Western blotting (Fig. 6E and F). To examine whether the blocking of Rab-mediated transport affected CSFV infection, SUVECs were transfected with Rab5(S34N) or Rab7(T22N). After transfection for 24 h, cells were infected with CSFV (MOI of 1), and viral genome copy numbers and viral titrations were determined at the indicated time points. We showed that CSFV proliferation was inhibited in Rab5(S34N)- or Rab7(T22N)-transfected cells (*P < *0.05) (Fig. 6G and H). As expected, we also observed that CSFV particles colocalized with Rab5 and Rab7 during invasion (Fig. 6I and Fig. 7J), suggesting that early/late endosomes are required for CSFV endocytosis and subsequent productive infection.

To investigate whether COP I-depleted cells exhibited any defects in trafficking to early or late endosomes, SUVECs were pretreated with inhibitors of BFA and GCA to disrupt COP I function, and the colocalization of the virus with Rab5 or Rab7 was examined using confocal microscopy after CSFV infection for 2 h. We observed a significant decrease in the number of CSFV particles colocalized with Rab7 (Fig. 6J) but not with Rab5 (Fig. 6I), suggesting that virus particles exhibit defective trafficking to late endosomes in COP I-depleted cells. We also revealed that CSFV core (C) and E2 proteins colocalized with the COP I subunit COPβ (Fig. 7), which indicated that COP I vesicles were used for CSFV transport. Together, these findings strongly suggest that COP I vesicles are involved in virion trafficking from early to late endosomes in CSFV entry into SUVECs.

### CSFV infection induces GBF1, ARF family, and COP I mRNA expression.

**FIG 8:**
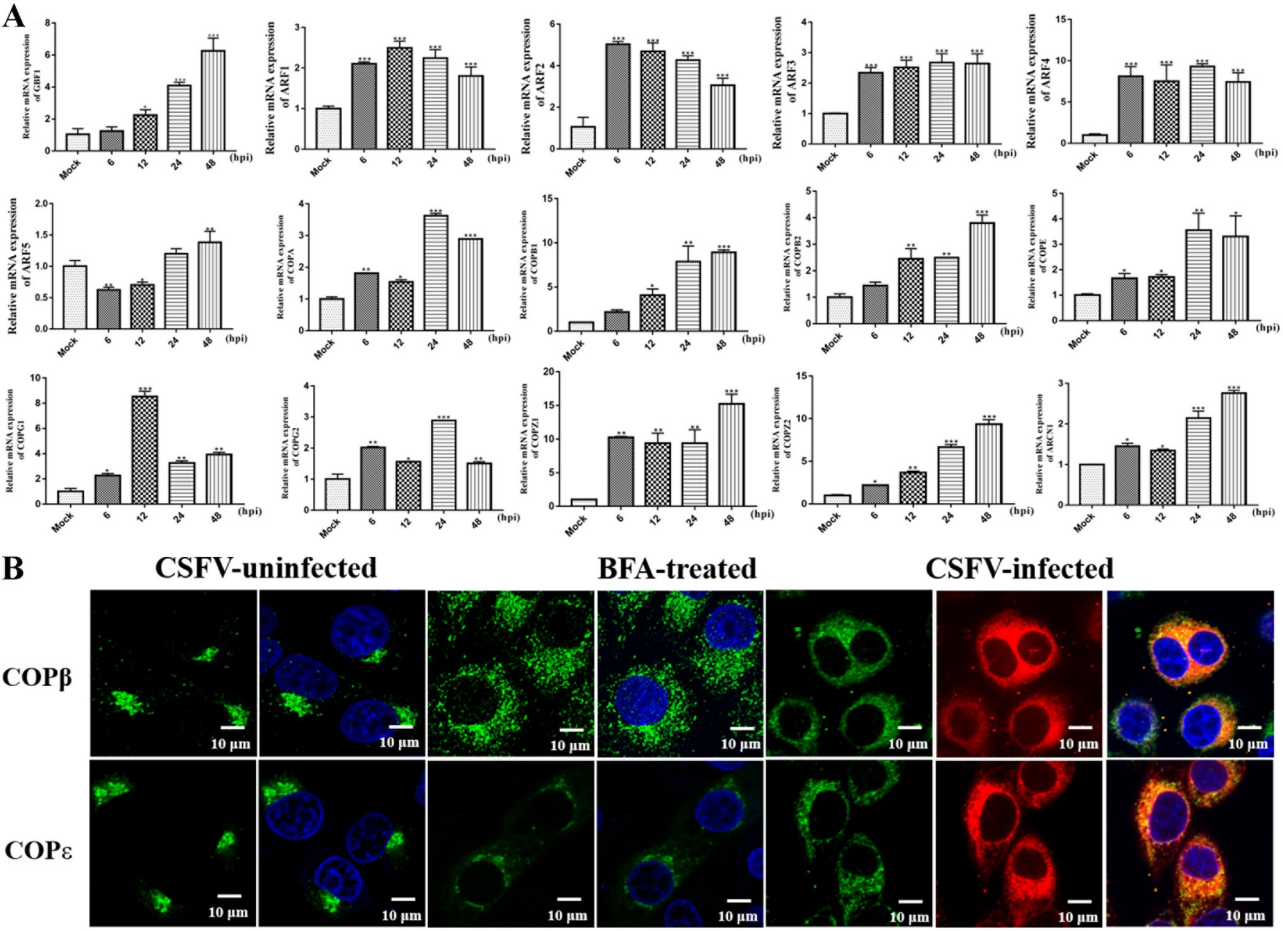
CSFV infection induces GBF1, ARF family, and COP I mRNA expression and disrupts the distribution of COP I-coated vesicles. (A) SUVECs were infected with CSFV (MOI of 1) for 6, 12, 24, and 48 h. Next, cells were collected for RNA extraction with TRIzol lysis at the indicated times, and the GBF1, ARF1, ARF2, ARF3, ARF4, ARF5, and COP I mRNA expression levels were measured by using RT-qPCR. (B) SUVECs were infected with CSFV (MOI of 1) for 24 h. Next, cells were fixed in 4% paraformaldehyde, and immunofluorescence staining was performed with the indicated antibodies. Cells were also counterstained with DAPI to label nuclei (blue).

Since the GBF1-ARF-COP I complex could regulate the proliferation of CSFV in SUVECs, whether CSFV infection of cells affects the expression of these host proteins is also worthy of attention. SUVECs were inoculated into 12-well plates and infected with CSFV at an MOI of 1, and the GBF1, ARF1 to ARF5, and COP I mRNA expression levels were measured using RT-qPCR. The results showed that CSFV infection significantly promoted GBF1, ARF1 to ARF4, and COP I mRNA expression at each time point (*P < *0.05), whereas the effect of CSFV on ARF5 mRNA expression showed inhibition at 6 and 12 h and then promotion at 48 h (Fig. 8A). Additionally, confocal laser microscopy revealed that the distribution of COP I-coated vesicles was disrupted in CSFV-infected cells (Fig. 8B). These results indicate that CSFV infection induces the expression of the GBF1-ARF-COP I complex and mediates the distribution of COP I-coated vesicles.

## DISCUSSION

In eukaryotic cells, the intracellular transport system is a complex and precise membrane structure composed of various organelles, cytoskeleton, and intracellular vesicles. The main function of the intracellular transport system is to accurately transfer the extracellular uptake of substances to various organelles or transfer substances synthesized in the ER to the target organelle or outside the cells. These special intracellular transport systems are indispensable for viruses to complete intracellular proliferation, including virus adsorption on the cell surface, invasion, genome replication, assembly, and the secretion of progeny viral particles (24). Our previous studies have revealed that the Rab1b-GBF1 axis, an important regulator of intracellular trafficking, was required for CSFV proliferation (17). Our present findings demonstrated that the Sec7 domain of GBF1 could rescue the inhibition of CSFV infection caused by BFA and GCA. This result revealed that the Sec7 domain is required for CSFV infection. The GEF activity of GBF1 is catalyzed by the Sec7 domain, which is responsible for activating class I and II ARFs (25). Therefore, we speculated that the activation of at least one BFA- or GCA-sensitive ARF family member is required for CSFV replication. Subsequent results showed that the overexpression of the ARF family could rescue the inhibition of CSFV infection caused by BFA and GCA. Intriguingly, ARF1 showed a better effect than other ARF members, suggesting that ARF1 is a key effector of the Sec7 domain of GBF1 in CSFV infection. We further showed that ARF1 with GBF1 Sec7 domain-dependently activates COP I to support CSFV invasion. Previous studies reported that the GBF1-ARF1-COP I axis was required for the replication of various viruses, including dengue virus (DENV) (13), HCV (26), and chikungunya virus (CHIKV) (27), which is consistent with our findings. However, Hazara nairovirus (HAZV) also utilizes COP I in an ARF1-independent manner to promote genome replication, possibly because the transport function of COP I-coated vesicles was not required for this step (28).

In addressing the mechanism of the GBF1-ARF1-COP I function in CSFV invasion, we observed that plasma membrane cholesterol was reduced due to a cholesterol-trafficking defect in cells caused by COP I depletion, and this led to a significant defect in CSFV invasion. We also showed that supplementation with exogenous cholesterol restored CSFV invasion caused by COP I inhibition. Cholesterol is synthesized in the smooth ER and leaves the ER rapidly toward the plasma membrane to regulate the invasion of viruses (29, 30). There seem to be two modes of cholesterol mechanisms involved in the virus invasion step. One mediates fusion between the virion and endosomes to support viral genome release into the cytosol, as in the cases of severe acute respiratory syndrome coronavirus 2 (SARS-CoV-2) (31), influenza A virus (IAV) (32), and porcine reproductive and respiratory syndrome virus (PRRSV) (33), whereas the other, which includes vaccinia virus (VACV) (34), human immunodeficiency virus (HIV) (35), and West Nile virus (WNV) (36), functions in a lipid raft-dependent manner and is involved in virus entry. Previously, studies have shown that cholesterol is required for CSFV infection, especially at the invasion step. Liang and colleagues reported that U18666A, which leads to cholesterol accumulation in the endosomal/lysosomal compartment through inhibition of cholesterol delivery to the membrane of late endosomes/lysosomes, inhibits CSFV fusion or uncoating (37). Yu and colleagues demonstrated that the depletion of plasma membrane cholesterol with methyl-β-cyclodextrin (MβCD), which is primarily used as a cholesterol-depleting reagent, could cause a failure of CSFV internalization (21). They also suggested that cholesterol was participating in CSFV invasion, possibly unrelated to lipid rafts (21). Previous research showed that the depletion of plasma membrane cholesterol by MβCD causes an increase in membrane tension, and high plasma membrane tension correlates with endocytosis inhibition (38, 39). The depletion of plasma membrane cholesterol or disruption of cholesterol trafficking results in changes in the fluidity, thickness, and intrinsic curvature of the membrane and affects the function of integral membrane proteins, including viral receptor or coreceptor conformation or distribution (40). Thus, it will be meaningful to reveal the role of cholesterol in virus invasion from the perspective of the relationship between cholesterol and the function or conformation of plasma membrane proteins. The GBF1-ARF1-COP I axis is an important part of the early secretion pathway of host cells (24). Moreover, it has been identified that COP I proteins mediate many additional effects beyond the well-studied trafficking roles within the early secretory pathways, including lipid homeostasis (41, 42), cholesterol transport (43, 44), and endosome maturation (45). However, the reason for cholesterol relocalization in COP I-depleted cells remains unclear. Defining the underlying mechanism of COP I in cholesterol trafficking will be the focus of our next study.

We also showed that the depletion of COP I impaired the colocalization of CSFV with the late endosome marker Rab7, suggesting that COP I depletion leads to a defect in CSFV invasion through inhibiting CSFV transport to late endosomes. Previous research indicated that COP I plays several roles in the life cycles of different viruses, including an involvement in endosome-dependent virus invasion, and COP I depletion perturbs cellular endocytic transport and thereby indirectly impairs vesicular stomatitis virus (VSV) entry (46). COP I depletion does not perturb influenza virus binding but also inhibits virus transport to late endosomes, as previously reported (14). One point worth noting is that CSFV invasion is sensitive to endosomal cholesterol, while its invasion defect in COP I-depleted cells is not due to the accumulation of endosomal cholesterol because cholesterol accumulation in COP I-depleted cells is different from U18666A, which leads to cholesterol accumulation in the endosomal/lysosomal compartment (47). These data reveal that COP I mediates virus invasion by regulating virion endosomal trafficking, while the role of COP I in the maturation of endosomes may provide an explanation for our results.

In summary, our current results support a model in which COP I assists CSFV to enter SUVECs in two different ways, depending on the GBF1-ARF1 function. On the one hand, the GBF1-ARF1-COP I axis mediates cholesterol trafficking to the plasma membrane to support CSFV entry. This way may play a major role in CSFV invasion since in COP I-inhibited cells, supplementation with exogenous cholesterol almost restored CSFV invasion. On the other hand, the GBF1-ARF1-COP I axis mediates CSFV transport from early to late endosomes during the entry steps.

## MATERIALS AND METHODS

### Cells and viruses.

The SUVEC, COPα-KO, and COPε-KO lines were maintained in medium 199 (Gibco, Grand Island, NY, USA) containing 10% FBS (Gibco) and a 1% penicillin-streptomycin solution (Sigma-Aldrich, St. Louis, MO, USA). Porcine kidney 15 (PK-15) cells were maintained in Dulbecco’s modified Eagle’s medium (DMEM) (Gibco) containing 10% FBS and a 1% penicillin-streptomycin solution. All cells were cultured at 37°C in a 5% CO_2_ incubator. The CSFV Shimen strain was obtained from the China Institute of Veterinary Drug Control (Beijing, China) and propagated in PK-15 cells.

### Plasmids and siRNA.

Cellular RNA was extracted from SUVECs using the AG RNAex Pro reagent (Accurate Biotechnology), and cDNA was synthesized using a PrimeScript first-strand cDNA synthesis kit (TaKaRa Bio). The swine ARF1 to ARF5 genes were amplified from cDNA using PCR and cloned into pEGFP-N1 or pCDH-CMV-513B vectors to obtain pEGFP-ARF1, pEGFP-ARF2, pEGFP-ARF3, pEGFP-ARF4, pEGFP-ARF5, and CMV-ARF1, respectively. The swine COPA and COPE genes were amplified from cDNA using PCR and cloned into pCDNA-3.1 vectors to obtain pcDNA-COPA and pcDNA-COPE. pcDNA-FLAG-GBF1, pcDNA-FLAG-GBF1(1–885), pcDNA-FLAG-GBF1(1–698), pcDNA-FLAG-GBF1-Sec7, ARF1-T31N, ARF1-Q71L, Rab5-S34N, and Rab7-T22N were constructed at Genecreate (Wuhan, China). The siRNA duplexes used in this study were siRab5 (catalog number sc-36344) and siRab7 (catalog number sc-29460). Plasmids pX458-COPA and pX458-COPE were constructed at GeneBiogist (Shanghai, China). The CRISPR/Cas9 knockout cell pSpCas9(BB)-2A-GFP vector was used to clone COPA and COPE single guide RNA (sgRNA) sequences between the BbsI sites. The primers used in this study are listed in Table 1.

**TABLE 1:** Primers used for plasmid construction

Primer	Sequence (5′–3′)
pEGFP-ARF1-F	CCCAAGCTTTGGGGAATATCTTTGCAAACCTCT
pEGFP-ARF1-R	CGGGATCCTCACTTCTGGTTCCGGAGCTGATTG

pEGFP-ARF2-F	CCCAAGCTTATGGGGAACGTTTTCGAAAAACTCT
pEGFP-ARF2-R	CGGGATCCTCACTTCTGGTTTTTGAGCTGGTTG

pEGFP-ARF3-F	CCCAAGCTTATGGGTAATATCTTTGGAAACCTCC
pEGFP-ARF3-R	CGGGATCCTCACTTCTTGTTTTTGAGCTGATTG

pEGFP-ARF4-F	CCCAAGCTTATGGGCCTCACCATCTCCTCCCTCT
pEGFP-ARF4-R	CGGGATCCTTAACGTTTTGAAAGCTCATTTGAC

pEGFP-ARF5-F	GCAGATATCCTAGCGCTTCGACAGCTCATGGGAC
pEGFP-ARF5-R	GGGGTACCCTAGCGCTTCGACAGCTCATGGGAC

CMV-ARF1-F	GGAATTCTGCCACCATGGGGAATATCTTTGCAAACCTCT
CMV-ARF1-R	ATAAGAATGCGGCCGCTCACTTCTGGTTCCGGAGCTGATTG

pCDNA-COPA	GGAATTCTGCCACCATGCTAACCAAATTCGAGACCAAGA
pCDNA-COPA	ATAAGAATGCGGCCGCTTAGCGAAACTGCAGAGGACTGATC

pCDNA-COPE	GGAATTCTGCCACCATGGCGCCACCGGCACCCGGCCCG
pCDNA-COPE	CGGGATCCGGCACTGGGAGCATACTGCAGCACC

sgCOPA	ATGATCAGACCATCCGAGTGTGG
sgCOPE	CTGGTCATGGAAGTAGATGGAGG

### Cell viability assay.

Cell viability was measured using the CCK-8 assay (catalog number CK04; Dojindo) according to the manufacturer’s instructions. Briefly, cells were seeded into 96-well plates and inoculated with or without Exo-1, BFA, GCA, or MβCD at the indicated concentrations for 72 h in an incubator at 37°C with 5% CO_2_. Next, the cell viability reagent was directly added and incubated with cells for another 1 h at 37°C in a shaker (60 rpm). The absorbance values were recorded at 450 nm using an Infinite M200pro system (Tecan, Männedorf, Switzerland).

### RNA extraction and RT-qPCR.

RT-qPCR was performed to measure the expression levels of the indicated mRNAs using the specific primers listed in Table 2. Total cellular RNA was extracted using the RNAex Pro reagent (catalog number AG21101; Accurate Biotechnology) and quantified using a NanoDrop One instrument (Thermo Fisher Scientific, Waltham, MA, USA). Next, cDNA was synthesized using the Evo Moloney murine leukemia virus (M-MLV) RT for PCR kit (catalog number AG11604; Accurate Biotechnology). CSFV RNA and mRNA expression levels were normalized to those of the housekeeping gene β-actin, estimated using the SYBR green premix pro *Taq* HS qPCR kit (catalog number AG11701; Accurate Biotechnology) according to the manufacturer’s protocol and tested using the CFX Connect real-time PCR detection system (Bio-Rad, Hercules, CA, USA). Data were analyzed using the 2^−ΔΔ^*^CT^* method.

**TABLE 2:** Primers used for RT-qPCR

Primer	Sequence (5′–3′)
ARF1-F	GACGCTGTTCTGCTTGTG
ARF1-R	TCCCTCATAGAGCCCATC

ARF2-F	ATCCCTACAATCGGTTTC
ARF2-R	TAGTTCTTCTCGGGCTTC

ARF3-F	TGATCGGGAGCGAGTAAA
ARF3-R	TCAGCAGCGTTCATAGCA

ARF4-F	CCTCACCATCTCCTCCCT
ARF4-R	CTTGACCACCAACATCCC

ARF5-F	CCCCACCATAGGCTTCAA
ARF5-R	AGTAGTGCCGCCACAGAG

GBF1-F	GACCCTAGAGGAGTTTCG
GBF1-R	AAGCAGCACATTCCATAC

COPA-F	TCAGAATGCCCTCTACCT
COPA-R	CTCCTTCCTCTTGTCCCT

COPB1-F	TCCTGTTCTGTCCGATTT
COPB1-R	TGTCTTCCTTGGTGCTAC

COPB2-F	ATTACTACAGTGGTGGCA
COPB2-R	GCTTAACAATGATGCTCC

COPG1-F	GAGGAATCGGGTGGAGGT
COPG1-R	GTGGGATCATTGGACTGAAA

COPG2-F	CGAGGAGTCTGGTACTGG
COPG2-R	TCTGTGGCTTCCGTAGTT

COPE-F	TGACCAACACCACCTTCC
COPE-R	CTTCTCACCACCCACAGC

ARCN1-F	GGGCGAGATGGAGGATTA
ARCN1-R	ATGGGAGTGGGATGGTGA

COPZ1-F	CCAGTGTCAAGGAGCAAA
COPZ1-R	TCAATGAGTCGAAGAGGC

COPZ2-F	GATGCTCATGTCCGTTCTCACCTG
COPZ2-R	CACGCCGCCATCCACAATGTG

CSFV-F	GAGAAGGACAGCAGAACTAAGC
CSFV-R	TTACCGCCCATGCCAATAGG

β-actin-F	CAAGGACCTCTACGCCAACAC
β-actin-R	TGGAGGCGCGATGATCTT

### Virus titration by an indirect IFA.

An indirect IFA was used for viral titration using a previously described method (17). Briefly, cells were seeded into 96-well plates and inoculated with CSFV-containing cell supernatants for 72 h in an incubator at 37°C with 5% CO_2_. Next, the cells were fixed with 4% paraformaldehyde (PFA) for 20 min at room temperature and washed with cold phosphate-buffered saline (PBS) three times. After fixation, the cells were permeabilized with 0.2% Triton X-100 for 10 min at room temperature and washed three times with PBS. After blocking with 3% bovine serum albumin (BSA) for 2 h, the cells were incubated with mouse anti-E2 CSFV antibody (1:200) (Ab-mart) at room temperature for 2 h. After three washes with PBS, the cells were incubated with goat anti-mouse IgG(H+L) (Alexa Fluor 594) antibody (1:200) (catalog number ab150116; Abcam) for 1 h at 37°C. Fluorescence-positive wells were observed and recorded using a fluorescence inversion microscope (Nikon, Tokyo, Japan). Viral titration was determined as the 50% tissue culture infectious dose (TCID_50_) per milliliter using the method proposed by Reed and Muench (48).

### Measurement of virus replication using an indirect IFA.

CSFV replication (Fig. 4B, Fig. 5C, and Fig. 7B) was measured using an indirect IFA according to a method described previously (17). Briefly, CSFV-infected cells or inhibitor-treated cells were fixed with 4% paraformaldehyde for 20 min at room temperature and washed three times with cold PBS. After fixation, the cells were permeabilized with 0.2% Triton X-100 for 10 min at room temperature and washed three times with PBS. After blocking with 3% BSA for 2 h, the cells were incubated with mouse anti-E2 CSFV antibody (1:200) (Ab-mart) at room temperature for 2 h. After three washes with PBS, the cells were incubated with goat anti-mouse IgG(H+L) (Alexa Fluor 594) antibody (1:200) (catalog number ab150116; Abcam) for 1 h at 37°C. Fluorescence-positive wells were observed using a fluorescence inversion microscope (Nikon, Tokyo, Japan).

### Overexpressing cell lines were established using lentiviruses.

ARF1-overexpressing lentivirus was produced as previously described (17). Briefly, overexpression plasmids with three other plasmids (pGag/Pol, pRev, and pVSVG) were cotransfected into HEK293T cells using Lipofectamine 3000 transfection reagent (catalog number L3000015; Thermo Fisher Scientific). After 16 h, the medium was replaced with advanced DMEM containing 2% FBS, 0.01 mmol/L cholesterol (catalog number C8667; Sigma-Aldrich), 0.01 mmol/L l-α-phosphatidylcholine (catalog number P443; Sigma-Aldrich), 1:1,000-diluted chemically defined lipid (catalog number 11905031; Invitrogen), and 4 mmol/L l-glutamine (catalog number G7513; Sigma-Aldrich). After 48 h, the supernatants containing the lentivirus were collected. Lentiviral titers were determined as the TCID_50_ per milliliter in HEK293T cells. Lentiviruses (MOI of 1) were used to infect SUVECs. After 12 h of infection, SUVECs were cultured in fresh medium with puromycin (5 mg/mL) (catalog number P8833; Sigma-Aldrich) to select stable cell lines. The cytomegalovirus (CMV) empty vector was treated as a control.

### Western blotting.

Cells were collected, total proteins were extracted using radioimmunoprecipitation assay (RIPA) lysis buffer containing 1% protease inhibitor cocktail (catalog number HY-K0010; MedChem Express), and the protein concentration was measured using a bicinchoninic acid (BCA) protein assay kit (catalog number 23227; Thermo Scientific). Next, the same amount of protein sample was separated using 8% or 10% SDS-PAGE, followed by transfer to polyvinylidene difluoride (PVDF) membranes (catalog number ISEQ00010; Merck Millipore). After blocking with 5% milk, the PVDF membranes were incubated with the following primary antibodies at room temperature for 2 h: mouse anti-FLAG antibody (1:5,000) (catalog number 66008-3-Ig; Proteintech), rabbit anti-ARF1 antibody (1:1,000) (catalog number AF2479; Beyotime), rabbit anti-COPα antibody (1:4,000) (catalog number ab192919; Abcam), rabbit anti-COPε antibody (1:4,000) (catalog number ab180946; Abcam), rabbit anti-Rab5 antibody (1:4,000) (catalog number ab18211; Abcam), rabbit anti-Rab7 antibody (1:4,000) (catalog number ab74906; Abcam), or rabbit antiactin antibody (1:5,000) (catalog number ab8227; Abcam). Subsequently, the PVDF membranes were incubated with horseradish peroxidase-conjugated goat anti-mouse IgG (catalog number SA00001-1; Proteintech) or goat anti-rabbit IgG (catalog number SA00001-2; Proteintech) secondary antibody (1:5,000) at room temperature for 1 h. Finally, the signal was detected using an enhanced chemiluminescence analysis system; β-actin served as an internal control protein.

### Binding and entry assays.

Drug-pretreated cells or COPα-KO and COPε-KO cell lines were infected with CSFV (MOI of 10) and cultured for 1 h at 4°C to allow virus binding without internalization, followed by washing with a precooled citrate buffer solution (pH 3) three times to remove unbound virions (binding assay). Cells were cultured in fresh culture medium at 37°C for another 2 h to allow virus internalization. After inoculation, the cells were washed with a citrate buffer solution (pH 3) to remove the noninternalized virions on the surface of cells, and the cells were then harvested after washing with precooled PBS three times (entry).

### Confocal microscopy.

To determine the ARF distribution (Fig. 2A and B), SUVECs were seeded onto glass coverslips in 35-mm cell culture dishes and cultured overnight. Cells were then transfected with the indicated plasmids (pEGFP, pEGFP-ARF1, pEGFP-ARF2, pEGFP-ARF3, pEGFP-ARF4, and pEGFP-ARF5) for 48 h and treated for 2 h with BFA. To determine Golgi structures (Fig. 3B), SUVECs were treated with Exo-1 (5 μmol/L) for 2 h. To visualize the colocalization of CSFV, Rab5, and Rab7 (Fig. 6I and J), SUVECs were pretreated with the inhibitors BFA (100 nmol/L) and GCA (300 nmol/L) for 8 h to disrupt COP I function and infected with CSFV (MOI of 10) for 2 h. The colocalization of the virus with Rab5 or Rab7 was examined using confocal microscopy. To visualize the colocalization of CSFV C and E2 with COPβ (Fig. 7), SUVECs were infected with CSFV (MOI of 10) for 12 h.

Cells were then washed with precooled PBS three times, fixed with 4% paraformaldehyde for 20 min, and permeabilized with 0.5% Triton X-100 in PBS for 10 min at room temperature. After three washes with PBS, the cells were blocked with 3% BSA in PBS for 2 h at room temperature. The cells were then incubated with the indicated primary antibodies overnight at 4°C. After three washes with PBS, the cells were incubated with goat anti-mouse IgG(H+L) (Alexa Fluor 594/488) antibody (1:200) for 1 h at room temperature in the dark. Subsequently, the cells were counterstained with 4′,6-diamidino-2-phenylindole (DAPI) to label cell nuclei (blue) at 37°C for 5 min and washed with cold PBS. Finally, images were captured using a confocal laser scanning microscope (LSM510 Meta; Zeiss, Germany).

### Cholesterol staining.

BFA (100 nmol/L)- and GCA (300 nmol/L)-treated SUVECs (for 8 h) and COPα-KO and COPε-KO cell lines were washed with precooled PBS three times and fixed with 4% PFA for 15 min at room temperature. Cells were then stained with 50 μg/mL filipin (Sigma) for 30 min at 4°C and analyzed using confocal microscopy.

### Cholesterol measurement.

BFA (100 nmol/L)- and GCA (300 nmol/L)-treated SUVECs (for 8 h) and COPα-KO and COPε-KO cell lines were collected and washed twice with ice-cold PBS. Cells were lysed, and the total cholesterol content was assayed using a Cholesterol/Cholesterol Ester-Glo assay kit from Promega (catalog number J3190) according to the manufacturer’s instructions.

### Statistical analysis.

All experiments were performed at least three times, and data were expressed as means ± standard deviations (SD). The data were analyzed by a *t* test with GraphPad Prism 6 software (GraphPad Software, Inc., La Jolla, CA, USA). A *P* value of <0.05 was considered significant.
